# Not All Distraction Is Bad: Working Memory Vulnerability to Implicit Socioemotional Distraction Correlates with Negative Symptoms and Functional Impairment in Psychosis

**DOI:** 10.1155/2014/320948

**Published:** 2014-02-27

**Authors:** Quintino R. Mano, Gregory G. Brown, Heline Mirzakhanian, Khalima Bolden, Kristen S. Cadenhead, Gregory A. Light

**Affiliations:** ^1^San Diego Veterans Affairs Healthcare System, San Diego, CA 92161, USA; ^2^VISN-22 Mental Illness, Research, Education and Clinical Center (MIRECC), VA San Diego Healthcare System, San Diego, CA 92161, USA; ^3^Department of Psychiatry, University of California, San Diego, School of Medicine, San Diego, CA, USA

## Abstract

This study investigated implicit socioemotional modulation of working memory (WM) in the context of symptom severity and functional status in individuals with psychosis (*N* = 21). A delayed match-to-sample task was modified wherein task-irrelevant facial distracters were presented early and briefly during the rehearsal of pseudoword memoranda that varied incrementally in load size (1, 2, or 3 syllables). Facial distracters displayed happy, sad, or emotionally neutral expressions. Implicit socioemotional modulation of WM was indexed by subtracting task accuracy on nonfacial geometrical distraction trials from facial distraction trials. Results indicated that the amount of implicit socioemotional modulation of *high* WM load accuracy was significantly associated with negative symptoms (*r* = 0.63, *P* < 0.01), role functioning (*r* = −0.50, *P* < 0.05), social functioning (*r* = −0.55, *P* < 0.01), and global assessment of functioning (*r* = −0.53, *P* < 0.05). Specifically, *greater* attentional distraction of *high* WM load was associated with *less* severe symptoms and functional impairment. This study demonstrates the importance of the WM-socioemotional interface in influencing clinical and psychosocial functional status in psychosis.

## 1. Introduction

Attentional impairments are commonly observed in psychosis [1]. A classic view of attentional distraction is that it reflects cognitive impairment, that is, reduced ability to accurately maintain information in the presence of task-irrelevant stimuli. Yet, there are real-world situations wherein attentional distraction is adaptive. Consider a dyadic social encounter wherein the communicatee's changing facial expressions appropriately disrupt the communicator's thoughts. Here, attentional distraction adaptively permits the communicator to modulate ongoing cognition and attend to changing facial expressions in the communicatee. In other words, effective and reciprocal social encounters are those that demonstrate flexibility whereby communicators are sensitive to the facial expressions of the communicatee and are capable of modulating ongoing thoughts to attend to the communicatee. The present study aimed to capture the adaptability of this everyday challenge and gather proof of concept evidence by examining implicit socioemotional modulation of working memory (WM) in relation to symptom severity and functional status in individuals with psychosis. We reasoned that individuals with relatively severe psychosis have a WM system that is less sensitive to the moment-to-moment modulation of socioemotional stimuli. Though individuals with psychosis have general cognitive impairments, the WM construct was initially targeted because it maps onto ongoing cognitive processes and its interaction with socioemotional modulation.

We utilized a WM paradigm [2] that assesses WM in the context of implicit facial distraction. In the original study, Mano and colleagues [2] found that task-irrelevant socioemotional stimuli disrupted WM in the intermediate load condition (i.e., 2 syllables) but not in the low or high WM load conditions (1 Syllable and 3 syllables, resp.). Such, implicit facial distraction was important to study because although individuals with psychosis demonstrate impairments in explicitly identifying facial emotions [3], many also demonstrate relatively intact implicit facial emotion processing [4–6], even for “emotionally neutral faces” [7, 8]. Load size was manipulated to determine whether associations among implicit facial disruption of WM, symptom severity, and functional status are load dependent. The present delayed match-to-sample task is in standard use to measure the rehearsal and maintenance aspect of WM and is commonly used to assess WM in the psychosis research literature [9].

Viewing implicit socioemotional modulation of WM as adaptive in certain real-world circumstances, we hypothesized that symptom severity and functional status in psychosis are related to attentional distraction of WM by task-irrelevant faces. Specifically, *greater* symptom severity and functional impairment would be associated with relatively *less* implicit facial disruption of WM.

## 2. Experimental Procedures

### 2.1. Participants

Twenty-one (7 female; mean age = 23 ± 9; mean years of education = 12 ± 2) individuals are diagnosed with schizophrenia, schizophreniform disorder, psychosis NOS, schizoaffective disorder, or an affective psychosis that was confirmed using the SCID-I [10]. Among these participants, 17 were in their first-episode of psychosis and four with chronic schizophrenia. All were right handed. Exclusion criteria were (a) neurologic disorders, (b) substance abuse in the past 1 month if it is the first episode, (c) substance abuse in the past 6 months if it is chronic schizophrenia, (d) lifetime history of substance dependence, (e) history of head injury with loss of consciousness greater than five minutes and/or posttraumatic amnesia, (f) cognitive impairment, and (g) medical illnesses associated with cognitive impairment.

### 2.2. Measures of Symptom Severity and Functional Outcome

Symptom severity was scored on the scales for the assessment of positive symptoms (SAPS [11]) and negative symptoms (SANS [12]). Functional status was assessed with the global functioning: social (GF: social [13]) and global functioning: role (GF: role [14]) scales, as well as the global assessment functioning (GAF) scale [15].

### 2.3. WM Paradigm

The WM paradigm, developed by Mano and colleagues [2], employed a 3 × 2 design with the within-subjects factors including *load size* (1 versus 2 versus 3 syllables) and *distraction* (faces (happy, sad, and emotionally neutral) versus nonfacial geometrical oval figure). Stimuli included (1) pronounceable nonwords, (2) human faces (Radboud Faces Database [16]), and a (3) nonfacial geometrical control. Each trial began with a cross (e.g., “+”) presented at central fixation for 1000 ms and was comprised of three sequential phases. In the first phase (encoding), participants were given one, two, or three syllables to subvocally read and memorize (2-second phase duration). Unbeknownst to participants, distracters were briefly presented (33 ms) immediately after the pseudoword presentation phase. Distracters were task-irrelevant and presented at fixation. A non-facial neutral backward mask immediately replaced distracters and filled the duration of the rehearsal phase. (Participants were told the backward mask was a rehearsal indicator.) During the second phase (rehearsal), participants were instructed to mentally rehearse the syllables presented in the first phase, with the rehearsal interval duration varying among 8–16 seconds in two-second increments. The third phase (recognition) consisted of a recognition test in which two sets of pseudowords were presented and the participant was instructed to indicate (using the keyboard number pad with dominant right hand) which set was from the first phase (4-second phase duration). The total duration of the computerized task lasted approximately 40 minutes, with two breaks given after every 36 trials. Stimulus presentation and behavioral recordings were controlled using E-Prime (Psychology Software Tools, Inc., Pittsburgh, PA). Accuracy and speed were stressed. Dependent variables were response latencies and percentage correct.

### 2.4. Posttask Facial Affect Recognition Test

Following completion of the task, participants were asked in an open-ended manner whether they “noticed anything in the task.” If a participant did not freely report detecting a face in the task, then they were given a debriefing statement. If participants reported detecting a face during the task, then they were given the facial affect recognition test prior to presentation of the debriefing statement. This test consisted of pictures of facial expressions representing each of the eight emotional expressions (angry, contemptuous, disgusted, fearful, happy, neutral, sad, and surprised) in the Radboud Faces Database [16]. Participants were instructed to circle three emotions potentially seen in the task to assess awareness of emotional expressions of facial distracters.

### 2.5. Data Analysis

Outliers were identified and removed at the individual level such that response latencies ±2.5 SD away from each participant's mean were removed from analyses. The primary aim was to assess associations among implicit socioemotional modulation of WM, symptom severity, and functional status. As such, contrast variables were created that demonstrate the effects of facial distractors on WM performance at each load condition (e.g., 1, 2, and 3 syllables). Effects of facial distractors on each load condition were collapsed across valence type (happy, sad, and emotionally neutral) and contrasted with the effect of geometrical distraction, producing contrast variables for each load condition. For example, 1-syllable WM accuracy in the context of happy, sad, and neutral facial distractors was averaged and then subtracted from 1-syllable WM accuracy in the context of geometrical distraction. This calculation produced a “1-syllable/facial distraction accuracy *minus* 1-syllable/geometrical distraction accuracy” variable, a calculation that was repeated for the 2 and 3 syllable conditions. Effects of facial distracters were collapsed across valence type (happy, sad, and neutral) because sensitivity to emotional and emotionally neutral faces is common in psychosis [4–8]. Notably, individual differences in WM capacity were controlled with contrast variables because capacity is represented on both sides of the contrast. Pearson correlations assessed associations among contrast variables, symptom severity, and functional status. Finally, a 3 (WM load) × 2 (Distraction) repeated-measures ANOVAs tested for main effects and factorial interaction, performed separately for accuracy and response latency data.

## 3. Results and Discussion

**Figure 1:**
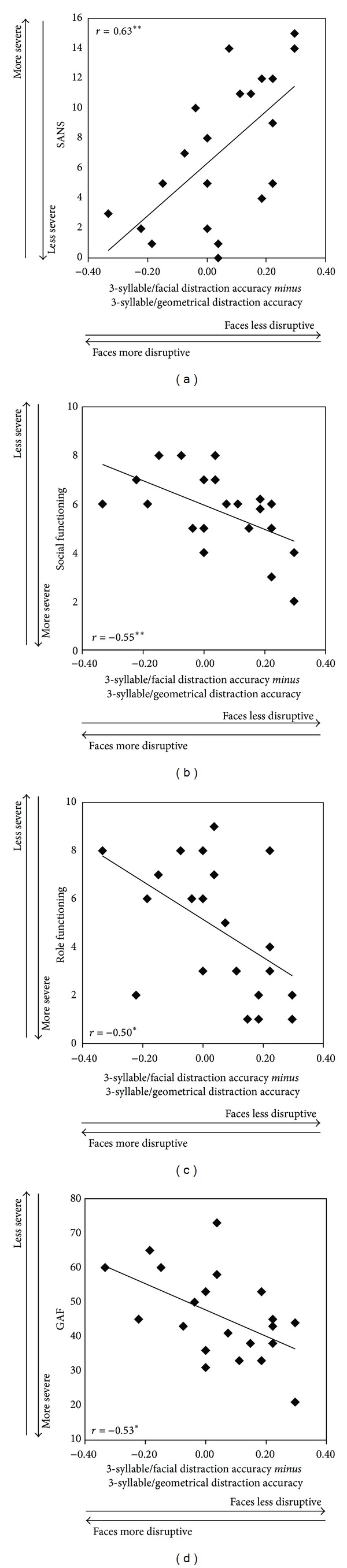
Correlations among contrast variables in the high working memory load condition (3 syllables) and measures of negative symptom severity and functional outcome.

Overall accuracy ranged from 68% to 100% (M = 88%; SD = 10%)—well above the 50% chance-level. Correlations among contrast variables and measures of negative symptom severity and functional status were statistically significant (Table 1) but only in the highest WM load condition (see Figure 1). The direction of the correlations in the highest WM load condition was such that patients showing the greatest disruption of WM performance due to facial distraction also experienced the least severe negative symptoms and the most intact functioning (Figure 1).

**Figure 2:**
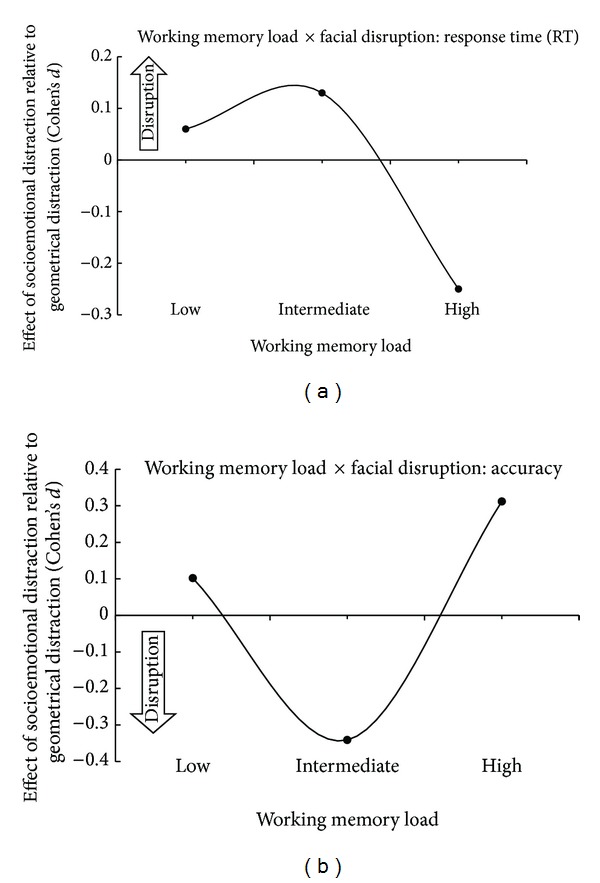
Modulation effects of all facial distracters are displayed relative to nonfacial geometric distracters. The zero axis value represents working memory performance in the context of nonfacial geometrical distracters. In (a) depicting response latencies relative to nonfacial geometrical distracters, positive effect sizes connote behavioral disruption while negative effect sizes connote behavioral facilitation. In (b) depicting accuracy relative to nonfacial geometrical distracters, positive effect sizes connote behavioral facilitation while negative effect sizes connote behavioral disruption.

Load had a significant effect on accuracy (*F*[2,19] = 23.079, *P* < 0.001; MSE = 0.01; *η* ^2^_*p*_ = 0.536) indicating that responses were most accurate for the 1-syllable condition (M = 94%; SD = 6%), intermediately accurate for the 2-syllable condition (M = 90%; SD = 11%), and least accurate for the 3-syllable condition (M = 80%; SD = 15%). In contrast, no significant main effect of distraction (*F*[2,19] = 0.906, *P* = 0.35; MSE = 0.008; *η* ^2^_*p*_ = 0.043) or load-by-distraction interaction (*F*[2,19] = 1.394, *P* = 0.260; MSE = .01; *η* ^2^_*p*_ = 0.065) was detected. Although the interaction failed to reach statistical significance, the partial eta-squared was of moderate strength and the interaction for response latencies was significant (*F*[1,20] = 3.676, *P* = 0.034; MSE = 20,022.396; *η* ^2^_*p*_ = 0.155).  Figure 2 displays the curvilinear relationship between WM load and socioemotional modulation for accuracy and response latencies.

All participants reported detecting a face during the task. Fifty-eight percent of the participants reported that they saw a happy face, 91% reported that they saw a neutral face, and 16% reported that they saw a sad face.

Results of the present study demonstrate that greater distraction of WM by task-irrelevant faces was associated with less severe symptoms and with more intact global mental-health functioning. These associations were load dependent and were observed only in the high WM load condition. How might these findings be explained? One account begins with the thesis that, because of their biological significance, faces are especially potent attractors of attention in the primate visual system [17]. Additionally, more normal facial processing is correlated with better social functioning in psychosis [18]. By implication, psychotic patients with intact face processing may be more distracted by irrelevant facial stimuli than psychotic patients with impaired facial processing and yet should function better.

Moreover, functional brain imaging studies have shown that not only that task-irrelevant emotional distraction can impair working memory, but also that disruptive emotional stimuli can activate limbic system sites while reducing activity in the dorsolateral prefrontal cortex [19, 20]. We have previously argued that as the effects associated with affective brain system responses become conscious, they compete with cognitive resources that otherwise would be dedicated to WM activity [21]. When WM load is small, resources may be shared without disrupting WM functioning. As WM load increases, the disruptive effects of facial distraction on WM performance appear until WM load approaches capacity, at which point high WM load processing maximizes capacity and deprives facial stimuli processing resources to cause disruption [2, 21]. This interactive process, particularly at the high WM load level, may be uniquely related to psychosocial functioning in disorders of psychosis. Though speculative, it is conceivable that some aspects of socioemotional processing are relatively impaired in psychosis because individuals with psychosis are engaged in high WM load processing during social encounters [21].

## 4. Conclusions

In a sample of individuals with psychosis, the amount of implicit socioemotional modulation of high WM load accuracy was significantly associated with negative symptoms, role functioning, social functioning, and global assessment of functioning. Specifically, greater attentional distraction of high WM load was associated with less severe symptoms and functional impairment. This study demonstrates the importance of the WM-socioemotional interface in influencing clinical and psychosocial functional status in psychosis.

## Figures and Tables

**Table 1:** Correlations among task variables (percent-correct), symptom severity, and functional outcome (*n* = 21).

Task variables	SANS	SAPS	Role	Social	GAF
Overall task	−.27	−.24	.54*	.20	.29
Overall 1-syllable	.23	−.17	.53*	.34	.36
Overall 2-syllable	−.10	−.18	.46*	.16	.20
Overall 3-syllable	−.28	−.27	.50*	.14	.28
1-syllable/facial distraction					
*minus* 1-syllable/geometrical distraction	−.11	−.13	.12	.18	.15
2-syllable/facial distraction					
*minus* 2-syllable/geometrical distraction	−.14	−.08	.12	−.08	.21
3-syllable/facial distraction					
*minus* 3-syllable/geometrical distraction	.63**	.35	−.50*	−.55**	−.53*
3-syllable/*happy* facial distraction					
*minus* 3-syllable/geometrical distraction	.59**	.27	−.54*	−.39	−.53*
3-syllable/*neutral* facial distraction					
*minus* 3-syllable/geometrical distraction	.60**	.28	−.50*	−.61**	−.46*
3-syllable/*sad* facial distraction					
*minus* 3-syllable/geometrical distraction	.49*	.40	−.30	−.45*	−.42

SANS: scale for the assessment of negative symptoms; SAPS: scale for the assessment of positive symptoms; role: global functioning: role; social: global functioning: social; GAF: global assessment of functioning.

*P ≤ 0.05; **P ≤ 0.01.
